# Risco de Fibrilação Atrial após Ablação de Flutter Dependente de Istmo Cavo-Tricuspídeo: Vale a Pena Fazer a Ablação da FA Simultaneamente?

**DOI:** 10.36660/abc.20190238

**Published:** 2020-05-22

**Authors:** Isabella Bianco, Gabriel Odozynski da Silva, Alexander Romeno Janner Dal Forno, Helcio Garcia Nascimento, Andrei Lewandowski, Elayne Pereira, André d’Avila

**Affiliations:** 1 Universidade do Sul de Santa Catarina PalhoçaSC Brasil Universidade do Sul de Santa Catarina, Palhoça, SC - Brasil; 2 Hospital SOS Cardio FlorianópolisSC Brasil Hospital SOS Cardio, Florianópolis, SC – Brasil

**Keywords:** Arritmias Cardíacas, Flutter Atrial, Condução, Ablação por Radiofrequência, Istmo Cavo-Tricuspídeo, Fibrilação Atrial/prevenção

## Abstract

**Fundamento:**

A ablação da fibrilação atrial (FA) e do *flutter* atrial dependente de istmo cavo-tricuspídeo (FLA-ICT) pode ser realizada simultaneamente quando as duas arritmias tenham sido registradas antes do procedimento. Entretanto, a melhor abordagem não é clara quando pacientes com FLA-ICT são encaminhados para ablação sem o registro prévio de FA.

**Objetivos:**

Avaliar a prevalência e identificar os preditores de ocorrência do primeiro episódio de FA após ablação de FLA-ICT em pacientes sem o registro prévio de FA.

**Métodos:**

Coorte retrospectiva de pacientes submetidos exclusivamente a ablação por cateter para FLA-ICT, sem registro prévio de FA. As características clínicas foram comparadas entre os grupos em que houve ocorrência de FA pós-ablação de FLA-ICT vs. sem ocorrência de FA. O nível de significância estatística adotado foi de 5%. Na análise de preditores, o desfecho primário avaliado foi ocorrência de FA após ablação de FLA-ICT.

**Resultados:**

De um total de 227 pacientes submetidos a ablação de FLA-ICT (110 com registro de FA e 33 sem seguimento adequado), 84 pacientes foram incluídos, dos quais 45 (53,6%) apresentaram FA pós-ablação. Não houve variáveis preditoras de ocorrência de FA. Os escores HATCH e CHA_2_DS_2_-VASC foram semelhantes nos dois grupos. As taxas de recorrência de FLA-ICT e complicações após a ablação foram de 11,5% e 1,2%, respectivamente.

**Conclusões:**

A ablação de FLA-ICT é eficaz e segura, mas 50% dos pacientes desenvolvem FA após ablação. Entretanto, ainda é incerto o papel da ablação combinada (FLA-ICT e FA) visando prevenção da ocorrência de FA. (Arq Bras Cardiol. 2020; [online].ahead print, PP.0-0)

## Introdução

O flutter atrial dependente de istmo cavo-tricuspídeo (FLA-ICT) é uma arritmia cardíaca comum, tratada de maneira eficaz e segura através de ablação por radiofrequência com taxas de sucesso e complicações respectivamente de 92-97% e 0,5-2,6%.^1-4^ Neste grupo de pacientes, aqueles com registro de fibrilação atrial (FA) antes da ablação do flutter apresentam taxa de recorrência de FA de 30 a 50% nos primeiros 30 meses^5,6^ e de até 82% dos pacientes nos 90 meses seguintes.^7,8^ Estima-se que a FA e o FLA-ICT sejam faces da mesma atriopatia, havendo por isso comum associação entre as duas arritmias. Por esta razão, advoga-se que pacientes com *flutter* atrial comum, que tenham registro de FA, sejam submetidos simultaneamente a uma ablação de FA e *flutter* durante o primeiro procedimento, diminuindo os riscos e custos do tratamento quando um segundo procedimento fosse realizado.

Nosso estudo tem por objetivos avaliar a prevalência e identificar os preditores de ocorrência de FA após ablação de FLA-ICT num subgrupo de pacientes sem o registro de FA antes da ablação do *flutter*. Idealmente, caso fosse possível identificar um perfil de risco para a ocorrência de FA após a ablação do FLA-ICT, uma abordagem combinada, incluindo a ablação das duas arritmias, poderia ser sugerida em pacientes com *flutter* atrial que ainda não apresentaram registro de FA.^6,9-11^

## Métodos

### Desenho do estudo e participantes

**Figura 1:**
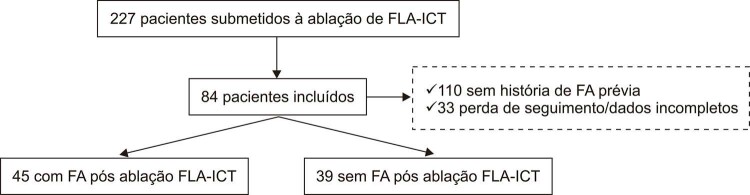
– Fluxograma de inclusão/exclusão do estudo: pacientes submetidos a ablação de FLA-ICT categorizados por ocorrência de FA pós-procedimento.

trata-se de uma coorte retrospectiva que avaliou pacientes de ambos os sexos ≥ 18 anos submetidos exclusivamente a ablação de FLA-ICT entre 2017 e 2018, no Hospital SOS Cárdio na cidade de Florianópolis-SC e Instituto de Cardiologia de Santa Catarina na cidade de São José-SC, com tempo de seguimento mínimo de 1 ano, e que não tivessem registro eletrocardiográfico de FA antes da ablação. Foram, portanto, excluídos da amostra pacientes com documentação eletrocardiográfica de FA anterior ao procedimento de ablação do FLA-ICT. A figura 1 ilustra o fluxograma de inclusão/exclusão dos participantes.

Este estudo foi aprovado pelo Comitê de Ética da Universidade do Sul de Santa Catarina (Unisul) sob o número de protocolo 79539517.1.0000.5369. Todos os procedimentos envolvidos neste estudo estão de acordo com a Declaração de Helsinki de 1975, atualizada em 2013, e a resolução CNS nº 466, de 12 de dezembro de 2012.

### Coleta de dados

Os pacientes incluídos no estudo, diagnosticados com FLA-ICT e submetidos à ablação por cateter, foram seguidos para ocorrência de FA após o procedimento *index*. As variáveis clínicas e associadas a comorbidades foram coletadas dos prontuários eletrônicos destes pacientes. A recorrência de FLA-ICT e a ocorrência de FA foram atestadas nos prontuários eletrônicos, através de eletrocardiograma e Holter de 24 horas, consultas de rotina, atendimentos de emergência e procedimentos de ablação.

### Protocolo de ablação de FLA-ICT

A ablação do FLA-ICT foi realizada sob anestesia geral. Foram realizadas duas punções na veia femoral direita, sendo posicionados cateteres decapolar deflectível no interior do seio coronário e cateter quadripolar de ablação com ponta de 8 mm. Em seguida, foi realizada a ablação (60W a 60°C por até 2 minutos) do ICT, iniciada junto à válvula tricúspide em direção à veia cava inferior às 6h na projeção oblíqua anterior esquerda (OAE), até a interrupção do *flutter* atrial. Após a interrupção da arritmia, foi observado duplo potencial atrial sobre a linha de ablação, com separação de pelo menos 100 milissegundos durante marcapasseamento contínuo do seio coronariano e da parede lateral do átrio direito para confirmação do bloqueio bidirecional, quando o procedimento foi, então, finalizado. Os pacientes foram mantidos em observação hospitalar por 24h após o procedimento, sendo orientados a retornar a seus respectivos médicos assistentes após a alta hospitalar.

### Análise estatística

As características clínicas e procedimentos foram comparados entre os grupos de pacientes em que houve ocorrência de FA pós-ablação de *flutter* vs. sem ocorrência de FA. Adotou-se uma amostra por conveniência (não-probabilística) durante o tempo de estudo, respeitando-se os critérios de inclusão/exclusão e tempo de seguimento.

Variáveis contínuas foram descritas como média e desvio padrão e comparadas utilizando teste-T de Student não-pareado (bicaudal), respeitando-se os critérios de normalidade pelo teste de Shapiro-Wilk. Variáveis categóricas foram descritas por número absoluto e porcentagens em relação à amostra total, sendo comparadas utilizando-se o teste de X^2^ ou Exato de Fischer. O nível de significância estatística adotado foi de 5%. Foi utilizada curva de Kaplan-Meier para evidenciar as taxas de recorrência ao longo do tempo de seguimento (por truncagem em 48 meses). Para avaliar os fatores preditores, foi realizada uma regressão logística com ocorrência de FA pós-ablação de *flutter* e não-ocorrência de FA pós-ablação de *flutter* como desfecho. Inicialmente todas as variáveis associadas com valor de p <0,20 na análise de regressão logística univariada foram inseridas nos modelos multivariados para ajuste final. Nenhum processo seletivo nos modelos multivariados foi realizado. A análise estatística foi realizada utilizando-se *IBM SPSS Statistics Editor software*, versão 22.0.

## Resultados

### Pacientes

Foi realizada ablação de *flutter* atrial em 227 pacientes entre 2017 e 2018 em dois centros de Santa Catarina, Brasil. Destes, 110 pacientes apresentavam registro prévio de FA, e em 33 pacientes não foi possível obter informação adequada durante o seguimento clínico. Portanto, 84 pacientes sem o registro de FA antes da ablação do FLA-ICT foram cadastrados para este estudo. Destes, durante um tempo de seguimento médio de 26±18 meses, 45 (53,6%) apresentaram FA pós-ablação. A Tabela 1 resume as características clínicas de pacientes com FA e sem FA após a ablação do FLA-ICT.

**Tabela 1:** – Características gerais de pacientes submetidos a ablação de flutter atrial, categorizados de acordo com ocorrência de fibrilação atrial durante seguimento

Variáveis	Ocorrência de FA (n = 45)	Sem ocorrência de FA (n = 39)	Valor de p
Idade (anos)	68,0 ± 12	66,4 ± 15	0,59
Sexo (masculino)	33 (73,2)	27 (69,2)	0,43
IMC	28,9 ± 4	29,7 ± 4,2	0,72
FEVE (%)	51,7 ± 14	54,8 ± 18	0,62
Diâmetro do AE (mm)	41,2 ± 7,8	42,2 ± 7,3	0,97
Comorbidades			
História de insuficiência renal	11 (24,4)	3 (7,2)	0,03
Dislipidemia	13 (28,9)	9 (23,1)	0,36
Insuficiência cardíaca	12 (26,7)	12 (30,8)	0,43
Hipertensão	32 (72,1)	22 (56,4)	0,12
Diabetes Mellitus	8 (17,8)	10 (25,6)	0,27
Doença vascular	16 (35,6)	9 (23,1)	0,15
AVC/AIT prévio	7 (15,6)	4 (10,3)	0,35
Medicações			
ACO prévio	23 (51,1)	21 (53,8)	0,33
DAA prévia	23 (51,1)	14 (35,9)	0,11
Escores			
HATCH	1 (1-3)	1 (0-3)	0,41
CHA_2_DS_2_-VASC	3 (2-4)	3 (1-4)	0,42

Valores com ± indicam a média e desvio padrão (idade, IMC, FEVE, diâmetro do AE); demais valores são apresentados em frequência simples e relativa. FA: fibrilação atrial; IMC: índice de massa corpórea; FEVE: fração de ejeção do ventrículo esquerdo; AE: átrio esquerdo; AVC: acidente vascular cerebral; AIT: ataque isquêmico transitório; ACO: anticoagulante oral; DAA: droga antiarrítmica; Teste t de Student e χ^2^ para amostras independentes. *p-valor indica diferença estatisticamente significativa ao nível de 5%.

A média de idade foi de 68 ± 12 anos no grupo com ocorrência de FA e de 66,4 ± 15 anos no grupo sem FA (p = 0,59). Indivíduos do sexo masculino representavam 73,2% dos pacientes no grupo com FA e 69,2% no grupo sem FA (p = 0,43). O IMC médio foi de 28,9 ± 4 kg/m^2^ grupo com FA e de 29,7 ± 4,2 kg/m^2^ no grupo sem FA (p = 0,72).

As comorbidades foram semelhantes nos dois grupos. A história de insuficiência renal e a hipertensão arterial sistêmica foram mais comuns no grupo com FA (24,4% FA x 7,2% [p = 0,03] e 72,1% FA x 56,4% [p = 0,12]). Não houve diferença entre os dois grupos no tocante a outras comorbidades, como dislipidemia, insuficiência cardíaca congestiva, diabetes mellitus, doença vascular e AVC/AIT (acidente vascular cerebral/ ataque isquêmico transitório) prévio e a utilização de anticoagulantes orais e drogas antiarrítmicas.

### Eficácia e segurança dos procedimentos

A taxa de recorrência de FLA-ICT após a ablação foi de 11,5%. A Tabela 2 resume os resultados dos procedimentos bem como a taxa de complicação. Houve rotura e embolização de ponta de uma bainha transeptal com curva fixa utilizada para estabilizar o cateter de ablação (taxa de complicação de 1,2%), que se alojou em um ramo distal da artéria pulmonar esquerda e não necessitou de intervenção cirúrgica para ser removida.

**Tabela 2:** – Resultado dos procedimentos: eficácia e segurança em 84 pacientes submetidos exclusivamente a ablação do istmo cavo-tricuspídeo para tratamento do *flutter* atrial comum

Evento	n (%)
Ocorrência de FA pós-ablação	45 (53,6)
Recorrência de flutter	10 (11,5)
Complicação da ablação do FLA-ICT	1 (1,2)

FA: fibrilação atrial; FLA-ICT: flutter atrial dependente de istmo cavo-tricuspídeo.

**Figura 2:**
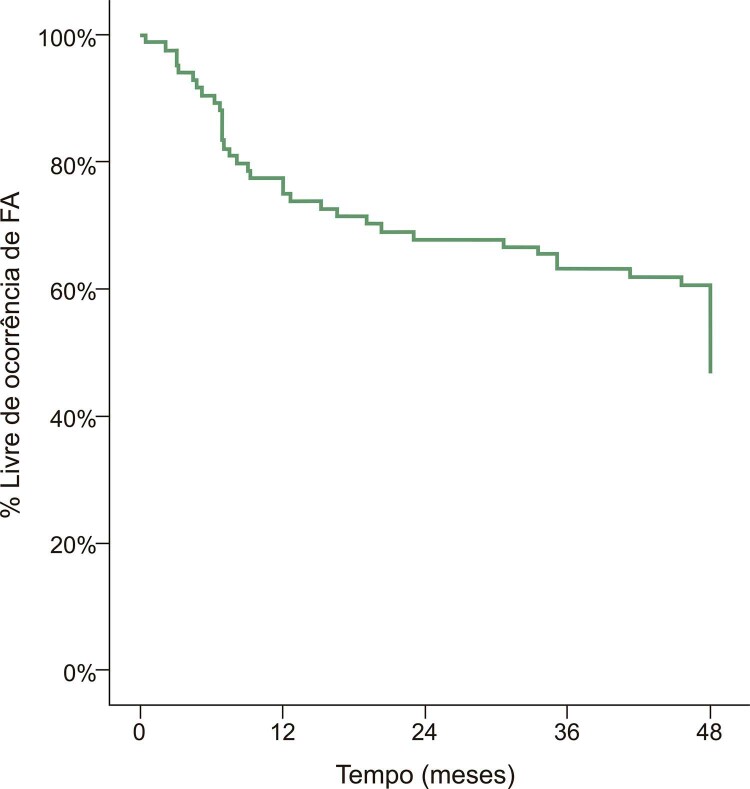
– Curva de Kaplan-Meier para ocorrência de FA pós-ablação de FLA-ICT.

A curva de Kaplan-Meier (Figura 2) mostra a ocorrência de FA de 53,6% após a ablação de FLA-ICT ao longo do tempo. Observa-se que a ocorrência de FA foi mais comum no primeiro ano após a ablação do FLA-ICT.

### Preditores de ocorrência de FA pós-ablação de FLA-ICT

**Figura 3:**
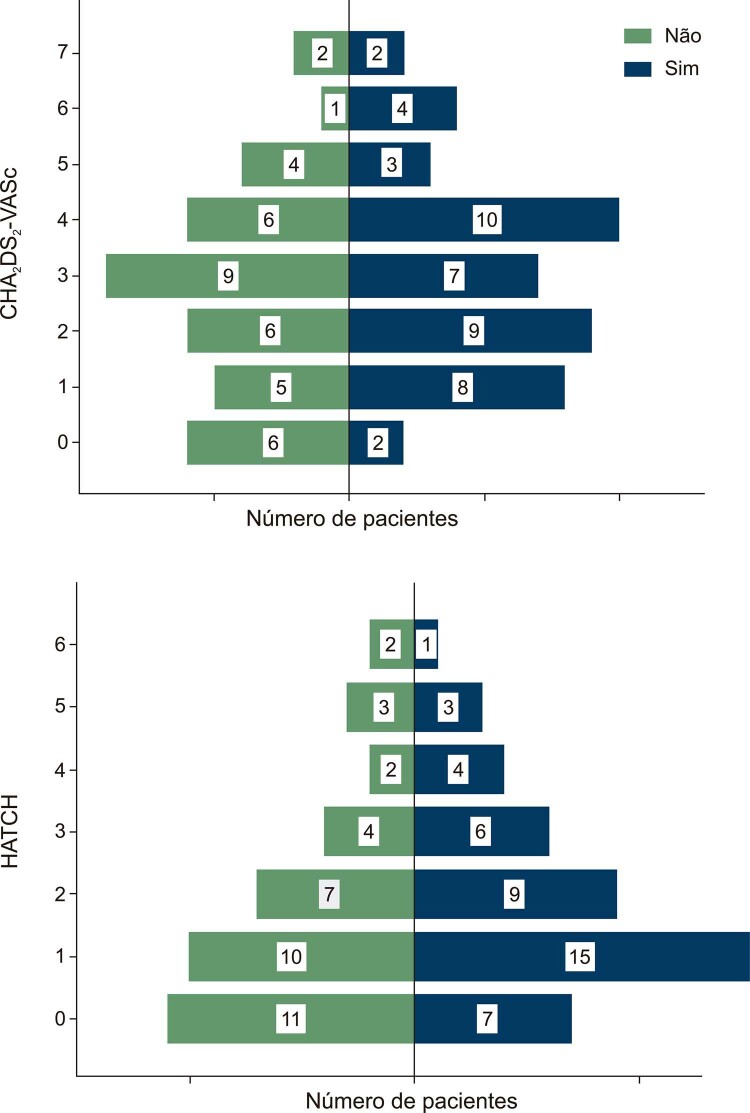
– Distribuição dos escores HATCH e CHA2DS2-VASC de acordo com ocorrência ou não de FA pós-ablação de FLA-ICT.

Na análise univariada foram encontrados preditores estatisticamente significativos para ocorrência de FA após o procedimento de ablação de FLA-ICT. As variáveis história de insuficiência renal (OR = 3,88 [IC_95%_ 0,99-15,1] p = 0,05) e hipertensão arterial sistêmica (OR = 2,15 [IC_95%_ 0,86-5,39] p = 0,10) foram inseridas nos modelos multivariados, no entanto não apresentaram significância estatística após ajuste do modelo (Tabela 3). A figura 3 mostra a distribuição dos escores HATCH e CHA2DS2-VASC de acordo com a ocorrência ou não de FA após o procedimento de ablação do FLA-ICT. Não houve diferença significativa entre os dois grupos. A distribuição dos valores do escore HATCH de acordo com a ocorrência ou não de FA pós-procedimento foi de 1 (1-3) no grupo com ocorrência de FA e de 1 (0-3) no grupo sem FA. Para o escore CHA2DS2-VASC, a distribuição foi de 3 (2-4) entre os pacientes com FA e de 3 (1-4) nos pacientes sem FA.

**Tabela 3:** – Análise univariada e multivariada de variáveis clínicas para a ocorrência de fibrilação atrial após ablação de flutter atrial dependente de istmo cavo-tricuspídeo

Variáveis	Análise Univariada			Análise Multivariada		
	OR	95% CI	Valor de p	OR	95% CI	Valor de p
Idade	1,01	0,98-1,04	0,38	-	-	-
Sexo	0,84	0,32-2,18	0,73	-	-	-
IMC	0,96	0,85-1,08	0,57	-	-	-
Diâmetro do AE	0,97	0,90-1,04	0,42	-	-	-
FEVE	0,99	0,96-1,02	0,82	-	-	-
História de insuficiência renal	3,88	0,99-15,1	0,05	3,12	0,89-14,2	0,10
ICC	0,66	0,25-1,73	0,40	-	-	-
HAS	2,15	0,86-5,39	0,10	1,98	0,78-5,04	0,15
DM	0,59	0,20-1,73	0,34	-	-	-
Vasculopatia	1,19	0,47-3,05	0,70	-	-	-
ACO prévio	1,32	0,56-3,13	0,51	-	-	-
DAA prévia	0,68	0,28-1,63	0,39	-	-	-

IMC: índice de massa corpórea; AE: átrio esquerdo; FEVE: fração de ejeção do ventrículo esquerdo; ICC: insuficiência cardíaca congestiva; HAS: hipertensão arterial sistêmica; DM: diabetes mellitus; ACO: anticoagulante oral; DAA: droga antiarrítmica. *p-valor indica diferença estatisticamente significativa ao nível de 5%.

## Discussão

Os principais achados deste estudo são: (1) a ablação de FLA-ICT é um procedimento eficaz e seguro, com baixas taxas de complicações (1,2%), (2) a ocorrência de FA após ablação de FLA-ICT é frequente (53,6%) em pacientes sem história prévia de FA, e (3) não houve critérios ou escores preditores para a ocorrência de FA após ablação de FLA-ICT.

### Ablação por radiofrequência de FLA-ICT

A ablação por radiofrequência dos circuitos arritmogênicos do FLA-ICT é um procedimento com altos índices de sucesso, com resultados superiores ao uso exclusivo de medicamentos antiarrítmicos.^9,12^ Dentre os efeitos colaterais conhecidos, em casos de recorrência do FLA-ICT, o uso de drogas antiarrítmicas como a propafenona pode facilitar a condução atrioventricular e aumentar a resposta ventricular com eventual instabilidade hemodinâmica. Além disso, não há melhora da qualidade de vida dos pacientes com tratamento por drogas antiarrítmicas, e 63% dos pacientes acabam sendo re-hospitalizados.13 Por esses motivos, a ablação por radiofrequência é recomendada como tratamento de escolha do FLA-ICT.

Em meta-análise recente, Pérez et al.^1^ descrevem taxa de recorrência de FLA-ICT de 10,6%, semelhante à descrita neste estudo, e taxas de complicações variam até 2,6%.^1,3^Pacientes com FLA-ICT submetidos à ablação com sucesso apresentam menor mortalidade e menor risco de AVC e eventos tromboembólicos em geral, comparativamente aos pacientes tratados com terapia medicamentosa exclusiva.^3^

No presente estudo, foram encontradas taxas de recorrência de FLA-ICT de 11,5% e complicações de 1,2%. Não houve nenhum evento embólico, derrame pericárdico ou óbito na amostra estudada apesar do longo tempo de seguimento dos pacientes.

### Ocorrência de FA pós-ablação de FLA-ICT

Em nosso estudo, foi encontrada taxa de ocorrência de FA pós-ablação de FLA-ICT de 53,6%. O aparecimento de FA após a ablação de FLA-ICT possui relevância clínica devido ao elevado risco de eventos tromboembólicos associados a essa arritmia, em especial o AVC. A presença de FA está associada a um aumento de quatro a cinco vezes no risco de AVC isquêmico. Os AVCs causados pela FA possuem maior mortalidade e provocam déficits funcionais mais severos.^14,15^Dessa maneira, não apenas os pacientes com FA têm maior risco de apresentar AVC, como também os AVCs que ocorrem nesses pacientes são mais graves e debilitantes. Em estudo envolvendo uma população de pacientes submetidos à ablação de FLA-ICT, a incidência de AVC ao longo de um seguimento médio de 40 meses pós-procedimento foi 4 vezes maior do que na população geral, e o único fator de risco identificado foi a ocorrência de FA pós-ablação de FLA-ICT.^16^ Por esse motivo, tendo em vista a alta incidência de FA nesta população, a interrupção do uso de anticoagulantes orais pode expô-los ao risco de eventos tromboembólicos, devendo ser avaliada individualmente levando-se em conta o CHA2DS2-VASC do paciente com flutter atrial tal como se utiliza em pacientes com FA.^17^

Assim sendo, uma parcela significativa dos pacientes continua sintomática em função da FA que passa a se manifestar clinicamente após a ablação de FLA-ICT. Um segundo procedimento de ablação, pode ser, então, necessário para o controle da FA. Apesar de o isolamento das veias pulmonares por ablação com radiofrequência, necessário para o tratamento da FA, ser um procedimento de maior complexidade, com maiores riscos e custos comparativamente à ablação de FLA-ICT, uma alternativa a ser considerada é a realização combinada de um único procedimento de ablação para eliminar ambas as arritmias, evitando-se uma segunda intervenção.^9,11^

Vale a pena realizar o isolamento das veias pulmonares simultaneamente à ablação do FLA-ICT em pacientes sem o registro prévio de FA?

Na ablação do FLA-ICT o eletrofisiologista busca a construção de uma linha de ablação na região do istmo cavo-tricuspídeo, no intuito de impedir e bloquear a condução do circuito macro-reentrante no átrio direito. Neste caso, o acesso dos cateteres de ablação ao átrio direito dá-se exclusivamente pela punção das veias femorais. A ablação de FA – por outro lado – é um procedimento de maior complexidade e duração que necessita de acesso ao átrio esquerdo por meio de uma punção transeptal (passagem dos cateteres do átrio direito ao esquerdo por punção pelo septo interatrial) para isolamento elétrico das veias pulmonares, geralmente responsáveis pelo gatilho da FA. O estudo REDUCE AF, envolvendo 216 pacientes, demonstrou que a ablação combinada de FLA-ICT + FA resultou em maior tempo livre de arritmia comparado ao grupo submetido à ablação de FLA-ICT apenas, especialmente nos pacientes com > 55 anos. Nesse subgrupo de pacientes, o número necessário para tratar (NNT) da estratégia de ablação combinada foi de 7, levando a uma redução de 14% no risco absoluto de ocorrência de FA.^11^

Em uma análise de custo-efetividade, um estudo canadense propõe que a ablação combinada de FLA-ICT + FA não apresenta benefícios do ponto de vista financeiro e de riscos envolvidos. Com uma taxa de incidência de FA de 33% ou menos, o custo médio da realização dos procedimentos separadamente (ablação de FLA-ICT apenas, e se necessário, ablação de FA no futuro) foi menor quando comparado à estratégia combinada. Para a ablação de FLA-ICT isolada, o risco médio também é menor, considerando-se que o risco da ablação de FA excede o risco da ablação de FLA-ICT em 25% ou mais. Deve-se considerar, no entanto, que os riscos, custos e complicações variam regionalmente assim como a incidência de FA pós ablação de FLA-ICT, sendo em nossa amostragem quase 2 vezes maior que o previsto em análises de custo-efetividade. Outra ressalva dá-se ao fato de as análises de custo-efetividade não ponderarem negativamente os eventos embólicos a longo prazo nos pacientes em que a FA passa a se manifestar. Em Santa Catarina, o custo médio da internação por AVC cardioembólico por FA chega a R$ 40.539,00 por paciente.18 Assim, os riscos e custos envolvidos na ablação combinada de FLA-ICT + FA não se justificariam em curto prazo; estudos a longo prazo investigando os benefícios relacionados à estratégia combinada serão necessários para sugerir o benefício da abordagem simultânea em pacientes sem o registro prévio de FA.19 Vale a pena destacar que o tratamento combinado é sempre realizado quando existe registro de FA em pacientes com FLA-ICT.

### Fatores preditores da ocorrência de FA

No presente estudo, nenhuma das variáveis ou escores analisados mostrou-se capaz de predizer a ocorrência de FA pós-ablação de FLA-ICT na população estudada. A literatura é divergente quanto aos achados de preditores para ocorrência de FA. Diferentes estudos encontraram variáveis clínicas como comorbidades, história prévia, duração do flutter atrial,^20^ variáveis ecocardiográficas e eletrocardiográficas como preditoras de FA.^6,10,21-23^ Apesar disso, Chinitz et al.,^5^ em estudo com 254 pacientes submetidos a ablação de FLA-ICT e seguimento de 30 ± 22 meses, não encontraram quaisquer variáveis preditoras para ocorrência de FA, mesmo dentre as mais comumente associadas à arritmia, corroborando nossos achados.

O escore HATCH foi proposto no intuito de predizer a progressão da atriopatia associada à FA, principalmente pela evolução clínica da FA paroxística à FA persistente. Em subanálises, o escore HATCH mostrou-se útil ao predizer ocorrência de FA em pacientes assintomáticos. Em nossa análise, não houve diferença entre os grupos quanto à ocorrência de FA pós-ablação de FLA-ICT relacionada ao escore HATCH.^24^ Além de predizer o risco de AVC na população portadora de FA, o escore CHA_2_DS_2_-VASC é sabidamente utilizado como preditor de morbimortalidade em diferentes contextos clínicos. No entanto, em nossa análise não houve significância estatística na predição da ocorrência de FA pós-ablação de FLA-ICT com base no CHA_2_DS_2_-VASC.

### Limitações

A primeira limitação associa-se ao caráter retrospectivo do estudo. Em segundo lugar, o tamanho limitado da amostra pode não ter sido suficiente para evidenciar diferenças entre os dois grupos (FA x não-FA) e identificar variáveis preditoras de FA pós-tratamento invasivo do FLA-ICT. Por fim, não foi realizado monitoramento para arritmias assintomáticas após a ablação de FLA-ICT, de modo que a real incidência de FA pode ter sido subestimada.

## Conclusões

Em nosso estudo, a ablação de FLA-ICT foi um procedimento eficaz e seguro. A FA apresentou alta incidência após ablação de FLA-ICT mesmo em pacientes sem história prévia de FA, independentemente das características clínicas dos pacientes. Não há dados suficientes para indicação de ablação combinada para tratamento do flutter atrial visando prevenção da ocorrência de FA. Estudos de maior seguimento serão necessários para documentar os reais benefícios de uma abordagem simultânea.
