# Supplementary material for: Risco de Fibrilação Atrial após Ablação de Flutter Dependente de Istmo Cavo-Tricuspídeo: Vale a Pena Fazer a Ablação da FA Simultaneamente?
Source: Arq Bras Cardiol. 2020 May 22;114(5):775–82. [Article in Portuguese] doi: 10.36660/abc.20190238 (PMC8386990; doi:10.36660/abc.20190238)
Supplement: Translation

# Translation: Risk of Atrial Fibrillation after Ablation of Cavotricuspid Isthmus-Dependent Atrial Flutter: Is Combined Ablation of Atrial Fibrillation Worthwhile?

**Authors:** Isabella Bianco, Gabriel Odozynski da Silva, Alexander Romeno Janner Dal Forno, Helcio Garcia Nascimento, Andrei Lewandowski, Elayne Pereira, André d’Avila

**Affiliations:** 1 Brazil Universidade do Sul de Santa Catarina, Palhoça, SC - Brazil; 2 Brazil Hospital SOS Cardio, Florianópolis, SC – Brazil

**Keywords:** Arrhythmias, Cardiac, Atrial Flutter, Conduction, Radiofrequency Ablation, Isthmus Cavo-Tricuspid, Arial Fibrillation/prevention

## Abstract

**Background:**

Simultaneous ablation of atrial fibrillation (AF) and cavotricuspid isthmus (CTI)-dependent atrial flutter can be performed when both arrythmias had been recorded before the procedure. However, the best approach has not been defined in case of patients referred for ablation with CTI-dependent atrial flutter, without history of AF.

**Objectives:**

To assess the prevalence and to identify predictors of the first episode of AF after ablation of CTI-dependent atrial flutter in patients without history of AF.

**Methods:**

Retrospective cohort of patients with CTI-dependent atrial flutter without history of AF undergoing catheter ablation. Clinical characteristics were compared between patients who developed AF and those who did not have AF after the procedure. Significance level was set at 5%. In the analysis of predicting factors, the primary outcome was occurrence of AF after CTI-dependent atrial flutter ablation.

**Results:**

Of a total of 227 patients undergoing ablation of CTI-dependent atrial flutter (110 with history of AF and 33 without adequate follow-up), 84 were included, and 45 (53.6%) developed post-ablation AF. The HATCH and CHA2DS2-VASC scores were not different between the groups. Recurrence rate of CTI-dependent atrial flutter and complication rate were 11.5% and 1.2%, respectively, after ablation.

**Conclusions:**

Although ablation of CTI-dependent atrial flutter is a safe and effective procedure, 50% of the patients developed AF after the procedure. However, the role of combined ablation (CTI-dependent atrial flutter plus AF) aiming at preventing AF is still uncertain. (Arq Bras Cardiol. 2020; [online].ahead print, PP.0-0)

## Introduction

Cavotricuspid isthmus (CTI)-dependent atrial flutter is a common cardiac arrhythmia, safely and effectively treated by radiofrequency ablation with success and complication rates of 92-97% and 0.5-2.6%,^1-4^ respectively. In this group of patients, those presenting atrial fibrillation (AF) before flutter ablation have an AF recurrence rate of 30-50% in the first 30 months^5,6^ and of up to 82% in the following 90 months.^7,8^It has been suggested that AF and CTI-dependent atrial flutter are manifestations of the same atrial disease, and thus are associated with each other. For this reason, it has been advocated that patients with common atrial flutter, and history of AF, should benefit from simultaneous ablation of AF and atrial flutter during the first procedure, reducing the risk and costs of treatment when a second procedure is needed.

The objective of our study was to assess the prevalence and to identify predictors of AF after ablation of CTI-dependent atrial flutter in a group of patients with no history of AF before flutter ablation. Ideally, if a risk profile for FA following CTI-dependent atrial flutter could be determined, a combined approach, including ablation of both arrhythmias, could be suggested in patients with atrial flutter and no history of AF.^6,9-11^

## Methods

### Study design and participants

This was a cross-sectional study that evaluated patients of both sexes aged 18 years or older, undergoing ablation of CTI-dependent atrial flutter between 2017 and 2018 at SOS Cardio Hospital in the city of Florianopolis, Brazil, and at Institute of Cardiology of Santa Catarina in the city of São Jose, Brazil, with a follow-up of one year or longer, without history of AF on electrocardiogram before ablation. Therefore, patients with electrocardiographic documentation of AF before ablation of CTI-dependent atrial flutter were excluded. The flowchart of patients’ inclusion and exclusion is illustrated in Figure 1.

**Figure 1:** – Flowchart of inclusion and exclusion of patients undergoing ablation of cavotricuspid isthmus-dependent atrial flutter categorized by occurrence of atrial fibrillation after the procedure.

This study was approved by the Ethics Committee of Universidade do Sul de Santa Catarina (Unisul) (approval number 79539517.1.0000.5369). All procedures involved in this study was conducted according the Helsinki Declaration, 1975, updated in 2013, and the 466 resolution of the Brazilian National Health Council (December 2012).

### Data collection

The patients included in the study, with diagnosis of CTI-dependent atrial flutter, undergoing catheter ablation, were followed for the occurrence of AF after the index procedure. Recurrence of CTI-dependent atrial flutter and occurrence of AF were confirmed by data collected from medical records – electrocardiogram, 24-hour Holter monitoring, routine consultations, emergency services and ablation procedures.

### Protocol of CTI-dependent atrial flutter ablation

Ablation of CTI-dependent atrial flutter was performed under general anesthesia. Two punctures were made in the right femoral vein, with placement of a decapolar deflectable 8mm ablation catheter. Ablation was then performed (60W at 60oC for up to 2 minutes), started near the tricuspid valve towards the inferior vena cava at six o’clock in a left-anterior oblique position, until interruption of the atrial flutter. When arrythmia was interrupted, double atrial potentials were observed on the ablation line, with periods of at least 100 milliseconds during continuous pacemaking of coronary sinus and lateral atrial wall for confirmation of bidirectional block and conclusion of the procedure. Patients were kept in observation for 24 hours after the procedure and instructed to consult their assistant physicians after hospital discharge.

### Statistical analysis

Clinical data and procedures were compared between the groups of patients with and without atrial flutter after the ablation procedure. A convenience (non-probabilistic) sample was used, according to inclusion and exclusion criteria, and time of follow-up.

Continuous variables were described as mean and standard deviation and compared using the unpaired, two-tailed Student’s t-test, according to normality of data distribution, assessed by the Shapiro-Wilk test. Categorical variables were described as absolute numbers and percentages and compared using the chi-square test of the Fisher’s exact test. Significance level was set at 5%. The Kaplan-Meier curve was used for analysis of recurrence rate during the follow-up period (truncation at 24 months). Predictive factors were assessed by logistic regression, with occurrence and non-occurrence of AF following atrial flutter ablation as outcomes. All variables associated with a p<0.20 in the univariate logistic regression analysis were included in the multivariate model for final adjustment.

No selection was applied in the multivariate models. Statistical analysis was performed using the IBM SPSS Statistics software, version 22.0.

## Results

### Patients

Atrial flutter ablation was performed in 227 patients between 2017 and 2018 at two centers in Santa Catarina, Brazil. Of these, 110 patients had a history of AF and 33 patients did not have enough clinical data. Therefore, 84 patients without history of AF before CTI-dependent atrial flutter ablation were enrolled in the study. During a mean follow-up of 26±18 months, 45 (53.6%) had AF after ablation. Table 1 summarizes clinical characteristics of patients with and without AF after ablation of CTI-dependent atrial flutter ablation.

**Table 1:** – Characteristics of patients undergoing ablation of atrial flutter, categorized according to occurrence of atrial fibrillation during the follow-up period

Variables	Occurrence of atrial fibrillation (n = 45)	Non-occurrence of atrial fibrillation (n = 39)	p-value
Age (years)	68.0 ± 12	66.4 ± 15	0.59
Sex (male)	33 (73.2)	27 (69.2)	0.43
Body mass index	28.9 ± 4	29.7 ± 4.2	0.72
LVEF (%)	51.7 ± 14	54.8 ± 18	0.62
Left atrial diameter (mm)	41.2 ± 7.8	42.2 ± 7.3	0.97
Comorbidities			
History of renal failure	11 (24.4)	3 (7.2)	0.03
Dyslipidemia	13 (28.9)	9 (23.1)	0.36
Heart failure	12 (26.7)	12 (30.8)	0.43
Hypertension	32 (72.1)	22 (56.4)	0.12
Diabetes mellitus	8 (17.8)	10 (25.6)	0.27
Vascular disease	16 (35.6)	9 (23.1)	0.15
Previous stroke/TIA	7 (15.6)	4 (10.3)	0.35
Medications			
OAC	23 (51.1)	21 (53.8)	0.33
AAD	23 (51.1)	14 (35.9)	0.11
Scores			
HATCH	1 (1-3)	1 (0-3)	0.41
CHA_2_DS_2_-VASC	3 (2-4)	3 (1-4)	0.42

Data expressed as mean ± standard deviation (age, body mass index, LVEF, left atrial diameter); or absolute and relative frequency; LVEF: left ventricular ejection fraction; TIA: transient ischemic attack; OAC: oral anticoagulants; AAD: antiarrhythmic drugs; Student’s t-test for independent samples; *p-value indicates statistically significant differences at a level of 5%

Mean age was 68±12 years in the group with AF and 66.4±15 years in the group without AF (p=0.59). In both groups, most patients were male (73.2% in the group with AF and 69.2% in the group without AF, p=0.43). Mean BMI was 28.9 ± 4 kg/m^2^ in the group with AF and 29.7 ± 4.2 kg/m^2^in the group without AF (p = 0.72).

Comorbidities were similar in both groups. History of renal failure and systemic arterial hypertension was more common in the group with AF (24.4% vs. 7.2% [p = 0.03] and 72.1% vs. x 56.4% [p = 0.12]). There was no difference between the groups regarding other comorbidities such as dyslipidemia, congestive heart failure, diabetes mellitus, vascular disease, stroke/transient ischemic attack, use of anticoagulants or antiarrhythmic drugs.

### Efficacy and safety of procedures

Recurrence rate of CTI-dependent atrial flutter was 11.5%. Table 2 summarizes the results of the procedure and the complication rate. There was rupture and embolization of the curved tip of the transseptal sheath used for stabilization of the ablation catheter (complication rate of 1.2%), that was lodged in the distal branch of the left pulmonary artery and was successfully removed without surgical intervention.

**Table 2:** – Efficacy and safety of ablation of cavotricuspid isthmus-dependent atrial flutter for treatment of common atrial flutter in 84 patients

Event	n (%)
Occurrence of post-ablation AF	45 (53.6)
Recurrence of atrial flutter	10 (11.5)
Complications	1 (1.2)

AF: atrial fibrillation.

The Kaplan-Meier curve (Figure 2) illustrates the occurrence rate of AF of 53.6% after ablation of CTI-dependent atrial flutter. The occurrence was more common in the first year after the procedure.

**Figure 2:** – Kaplan-Meier analysis of occurrence of atrial fibrillation after ablation of cavotricuspid isthmus-dependent atrial flutter.

### Predictors of AF after ablation of CTI-dependent atrial flutter

The univariate analysis revealed statistically significant predictors for the occurrence of AF after ablation of CTI-dependent atrial flutter. The variables history of renal failure (OR = 3.88 [95%CI 0.99-15.1] p = 0.05) and systemic arterial hypertension (OR = 2.15 [95%CI 0.86-5.39] p = 0.10) were included in the multivariate models, but did not show statistical significance after adjustment of the model (Table 3). Figure 3 shows the distribution of the HATCH and CHA_2_DS_2_-VASC scores according to the occurrence or not of AF after CTI-dependent atrial flutter ablation. No difference was found between the two groups. Distribution of the HATCH score values by occurrence or not of AF after ablation was 1 (1-3) in the group with AF and 1 (0-3) in the group without AF. Distribution of the CHA_2_DS_2_-VASC score values was 3 (2-4) and 3 (1-4) in patients with and without AF, respectively.

**Table 3:** – Univariate and multivariate analysis of clinical variables for occurrence of atrial fibrillation after ablation of cavotricuspid isthmus-dependent atrial flutter

Variables	Univariate analysis			Multivariate analysis		
	OR	95% CI	p-value	OR	95% CI	p-value
Age	1.01	0.98-1.04	0.38	-	-	-
Sex	0.84	0.32-2.18	0.73	-	-	-
Body mass index	0.96	0.85-1.08	0.57	-	-	-
Left atrial diameter	0.97	0.90-1.04	0.42	-	-	-
LVEF	0.99	0.96-1.02	0.82	-	-	-
History of renal failure	3.88	0.99-15.1	0.05	3.12	0.89-14.2	0.10
CHF	0.66	0.25-1.73	0.40	-	-	-
SAH	2.15	0.86-5.39	0.10	1.98	0.78-5.04	0.15
Diabetes mellitus	0.59	0.20-1.73	0.34	-	-	-
Vascular disease	1.19	0.47-3.05	0.70	-	-	-
OAC	1.32	0.56-3.13	0.51	-	-	-
AAD	0.68	0.28-1.63	0.39	-	-	-

LVEF: left ventricular ejection fraction; CHF: congestive heart failure; SAH: systemic arterial hypertension; OAC: oral anticoagulants; AAD: antiarrhythmic drugs. *p-value indicates statistically significant differences at a level of 5%

**Figure 3:** – Distribution of HATCH and CHA2DS2-VASC scores according to the occurrence (yes/no) of atrial fibrillation after ablation of cavotricuspid isthmus-dependent atrial flutter.

## Discussion

The main findings of the present study were (1) ablation of CTI-dependent atrial flutter is an effective and safe procedure, with low complication rates (1.2%); (2) AF is a frequently occurring complication (53.6%) in patients without history of AF; and (3) no criterion or predictive score for AF after ablation of CTI-dependent atrial flutter was identified.

### Ablation of CTI-dependent atrial flutter by radiofrequency

Ablation of arrhythmogenic circuits of CTI-dependent atrial flutter using radiofrequency is associated with high success rates, superior to the exclusive use of antiarrhythmic drugs.^9,12^Among the known side effects, in case of recurrence of CTI-dependent atrial flutter, the use of antiarrhythmic drugs like propafenone may facilitate the atrioventricular conduction and increase ventricular response, with possible hemodynamic instability. Besides, the quality of life of patients treated with antiarrhythmic drugs is not improved, and 63% of patients require readmission.^13^ Therefore, radiofrequency ablation is recommended as the treatment of choice for CTI-dependent atrial flutter.

In a recent meta-analysis, Pérez et al.^1^ reported a recurrence rate of CTI-dependent atrial flutter of 10.6%, similar to that found in the present study, and complication rates of up to 2.6%.^1,3^ Patients with CTI-dependent atrial flutter undergoing successful ablation showed lower mortality and lower risk of stroke and thromboembolic events, compared with patients treated only with drug therapy.^3^

In the present study, we found recurrence rate of CTI-dependent atrial flutter of 11.5% and complication rate of 1.2%. No patient had embolic event or pericardial effusion, and no patient died despite the long period of follow-up of the study sample.

### Occurrence of AF after ablation of CTI-dependent atrial flutter

In our study, recurrence rate of AF after ablation of CTI-dependent atrial flutter was 53.6%. This is of clinical significance, due to the high risk of thromboembolic events associated with this arrhythmia, particularly stroke. The presence of AF is associated with 4-5 times greater risk of developing ischemic stroke. Stroke caused by AF has been associated with higher mortality and more severe functional deficits.^14,15^ Thus, patients with AF are not only at greater risk of developing stroke, but also of having more severe disease, with more debilitating complications. In a study on patients undergoing ablation of CTI-dependent atrial flutter, the incidence of stroke during a mean follow-up of 40 months after the procedure was four times greater than the general population, and the only risk factor identified was occurrence of AF after ablation of CTI-dependent atrial flutter.^16^ For this reason, considering the high incidence of AF in this population, discontinuation of oral anticoagulation may expose them to the risk of thromboembolic events and hence should be considered individually, considering the CHA2DS2-VASC score of the patient with atrial flutter, just as with patients with AF.^17^

Therefore, a significant number of patients remains symptomatic due to the development of AF after CTI-dependent atrial flutter ablation. A second ablation procedure may be then necessary for the control of AF. Although isolation of pulmonary veins by radiofrequency ablation (required in the treatment of AF) is a more complex procedure, with higher costs and risks compared with CTI-dependent atrial flutter ablation, an alternative may be to treat both arrhythmias using a combined procedure, thus avoiding a second intervention.^9,11^

Is it worth to perform isolation of the pulmonary veins simultaneously with ablation of CTI-dependent atrial flutter in patients with history of AF?

In the ablation of CTI-dependent atrial flutter procedure, the electrophysiologist makes an ablation line in the cavo-tricuspid isthmus area, to prevent or block the macroreentrant circuit in the right atrium. In this case, the access of the ablation catheters to the right atrium occurs exclusively by puncture of the femoral veins. Ablation of AF ablation, in turn, is a more complex and time-taking procedure that requires the access to the left atrium by transseptal puncture (passage of the catheters from the right to the left atrium by puncture of the interatrial septum) for electrical isolation of the pulmonary veins, generally responsible for the triggering of AF. The REDUCE AF study, involving 216 patients, showed that combined ablation of CTI-dependent atrial flutter plus AF resulted in a longer arrhythmia- free interval compared with the CTI-dependent atrial flutter ablation alone, especially in >55 age patients. In this subgroup of patients, the number needed to treat (NNT) was seven, with an absolute risk reduction in AF occurrence of 14%.^11^

Using a cost-effectiveness analysis, a Canadian study proposed that the combined ablation (CTI-dependent atrial flutter plus AF) does not provide financial and risk benefit. With an incidence rate of AF of up to 33%, the mean cost of performing the procedures separately was lower than the combined strategy. In addition, when performed alone, the mean risk of ablation of CTI-dependent atrial flutter is lower, since the risk of AF ablation exceeds the risk of CTI-dependent atrial flutter ablation by 25% or more. One should consider, however, that risk, cost and complication rates vary regionally, like the incidence of AF after ablation of CTI-dependent atrial flutter, which was twice greater than that predicted in cost-effectiveness studies. Also, cost-effectiveness analysis usually does not consider the negative long-term impact of embolic events in patients with new onset AF. In the state of Santa Catarina, the mean cost of hospitalization due to cardioembolic stroke with AF reaches BRL 40,539 per patient.^18^ Thus, the risks and costs involved in the combined ablation procedure (CTI-dependent atrial flutter and AF) would not be justifiable in short term; instead, long-term studies investigating the benefits of combined procedures in patients without history of AF are needed.^19^ It is worth highlighting that the choice for the combined therapy is always made for patients with CTI-dependent atrial flutter and history of AF.

### Predicting factor for AF

In the present study, none of the variables or scores analyzed was able to predict the occurrence of AF after ablation of CTI-dependent atrial flutter in the study population. The literature about predictors for the occurrence of AF is inconclusive. Different studies have described clinical variables such as comorbidities, previous history of AF, duration of atrial flutter,^20^ echocardiographic and electrocardiographic variables as predictors of AF.^6,10,21-23^ On the other hand, Chinitz et al.,^5^in a study with 254 patients undergoing ablation of CTI-dependent atrial flutter, followed-up for a mean of 30 ± 22 months, did not find any predictors for AF, even among those more commonly associated with arrhythmia, corroborating our findings.

The HATCH score has been proposed to predict the progression of AF from paroxysmal to persistent AF. In sub-analyses, the HATCH was useful in predicting the occurrence of AF in asymptomatic patients. In our analysis, no difference was observed between the groups in the occurrence of AF after ablation of CTI-dependent atrial flutter according to the HATCH score.^24^ In addiction to predicting the risk of stroke in patients with AF, the CHA_2_DS_2_-VASC score is known to be used for prediction of morbidity and mortality in different clinical settings. However, in the present study, the score did not show statistically significance in predicting AF after ablation CTI-dependent atrial flutter.

### Limitations

The first limitation of the study is its retrospective nature. Second, the limited sample size may have prevented the detection of significant differences between the two groups (AF vs. non-AF) and identify predicting variables of AF after invasive treatment for CTI-dependent atrial flutter. Finally, we did not monitor asymptomatic arrythmias after ablation of CTI-dependent atrial flutter, and hence the real incidence of AF may have been underestimated.

## Conclusions

In our study, ablation of CTI-dependent atrial flutter was an effective and safe procedure. There was a high incidence of AF after the ablation procedure, even in patients without history of AF, and regardless of clinical characteristics of the patients. There is not enough evidence to recommend combined ablation for treatment of atrial flutter aiming at preventing the occurrence of AF. Studies with longer follow up are needed to determine the real benefits of simultaneous ablation.
